# Ophthalmia Neonatorum

**Published:** 1874-01

**Authors:** J. C. Bishop


Vol. IV.—JANUARY, 1874.—No. 1.

### Original Communications.

### "OPHTHALMIA NEONATORUM."

BY J. C. BISHOP, M.D.

*Read before the Meigs County Medical Association, at Middleport, O., March* 10, *A.D.* 1873.

Gentlemen,—My apology for presenting so brief an outline of the affection called "Ophthalmia Neonatorum," must be that I regard the disease with something as of more akin to importance than the books seem willing to accord; and I believe, too, that it does not occupy as important a place in works on ophthalmology as it deserves. As a class, these works do but little more than mention it, which I conceive is hardly just, both on account of the uneasiness it occasions the parents and friends, and of the sequellæ which are occasionally observed. As its name implies, it is an affection of young children, usually making its appearance during the first three or four weeks, and may appear on the first, second or third day after its birth, and we see the disease usually within the first week. The first symptom usually observed is, that the little patient keeps its eyes almost constantly closed; when, however, it does open them, it is only for an instant, and immediately closes them again, while the integument supra-orbital is wrinkled, as if pain were present; next, the eyelids at their edges are more or less tumefied or swollen, and red, and hot. When the child sleeps, they are glued together with a discharge, the super-secretion of the meibomian glands, until it is necessary to soften it before even an examination can be had, or the eyes at all opened. When they are opened, the palpebral conjunctivæ is seen to be red and spongy, while the sclerotic is nearly normal. Usually, the disease makes its appearance in one eye, and in a day or two the same phenomena is observed in the other. In the last eye, however, the disease is hardly so intense, owing to the fact that treatment is commenced before it has time to proceed so far in its course. The tumefaction which was first noticed at the edges soon involves the entire palpebral surface, venting itself most completely on the superior. At this time is established a discharge sero-muculent and whitish in character, which will, when the lids are separated, gush out and run down the cheeks; the lids being spasmodically closed, and their edges glued together by the discharge above mentioned, accumulates in the conjunctivæ space, its escape being thus effectually prevented.

If the conjunctivæ of the scleroticæ be at this time examined, it will be found red and infected even to the margin of the cornea. We notice, as the disease proceeds, that the palpebræ become more swollen and of a dark-purplish or brownish color, the temperature over and around the orbit somewhat increased, and it has a smooth, glistening appearance, and general febrile symptoms are noticed; the child nurses very little, is restless and fretful, and even sometimes refuses to take the breast at all, and, as a consequence, presents the languid, pinched appearance you would expect from deficient nutrition, and has more or less irritative fever. The upper lid being most swollen, sometimes will overlap the lower, and become additionally irritated by the presence on the mucous surface of the little lower lashes. The tissues surrounding the eye in children of this tender age being very lax, according to Soelberg Wells, page 53, serous infiltration is generally considerable, even in the milder cases.

When the disease has reached its acme, if you separate the lids, the discharge is changed in character from a thin sero-muculent to a thick puriform, and readily escapes. There is considerable engorgement of the vessels with blood, which, as it were, puffs up the conjunctivæ of the palpebral sinuses, and looks sarcomatous, and protrudes, which is a fruitful source of ectropion. This is especially liable from crying, as the muscles of the orbit are contracted thereby.

At this time the chemosis, which is always present to greater or less degree, is quite readily observed, and the lachrymal caruncle and semi-lunar fold are very much enlarged, and when this is present, it is not unusual by any means to have a slight discharge of blood from the conjunctival surface. Up to this time the cornea may have been entirely unaffected, or at most have presented a hazy or dim appearance, unless the chemosed condition have existed for a time, when it will be more or less injured, and even go on to ulceration, abscess, or even destructive purulent infiltration. When this is the case, the discharge (which, it will be remembered, has become purulent) becomes ichorous, and the swelling and tension of the lids somewhat diminishes.

During the presence, or rather the continuance, of such local manifestations as have been mentioned, the little patient continues to be uneasy and fretful or restless, and gives all the evidence such little sufferers can of the presence of intense pain; indeed, the symptoms are *a priori* vouchers of great suffering. The mouth, according to Jones, is often apthous, while I have seen more or less intestinal irritation, and at *times, constipated* bowels.

It has been asserted that those children are most liable to ophthalmia who are prematurely born, are weakly, or twins. This statement perhaps lacks confirmation. In fact, in the limited number of cases I have seen, the opposite obtained. As might be supposed, the causes of "Ophthalmia Neonatorum" are somewhat various. Among the most frequent causes are placed exposure to a strong light soon after birth, or a draught of cold air, soapsuds and spirits, which are often used in washing the child; contact during birth with a leucorrhœal or gonorrhœal discharge; the application of sponge, linen or other material, which having been used, and to which a particle of the *materies morbi* adheres; and in fact, the disease often appears when it is impossible to assign any cause for it, and when the medical attendant is left only his imagination to aid in search for the cause. While its propagation would seem to take place *in distans* as well as *per contactum*, though not so readily, especially in hospital wards, where a large number of children are crowded together. Children taking it *in distans* would not certainly be impossible, if we accept the cryptogamic or germ theory of disease. Dr. Cederschjold, of Stockholm, found, as quoted by Jones, ophthalmia neonatorum to occur in twenty out of one hundred and thirty-seven, or one in seven infants, the mothers of whom were affected with a vaginal discharge; and in ten out of one hundred and eighty-one, or one in eighteen, the mothers of whom were not affected—which also shows that nearly three times as many cases occur in children the mothers of whom had a discharge as the contrary; thus conclusively showing that the presence of a vaginal discharge is a fruitful source of the disease. However, it does not necessarily follow that because the mother has a discharge from the vagina, either leucorrhœal or gonorrhœal, that the child will have an ophthalmia, for cases undoubtedly occur in which children born under such circumstances do *not* have the disease. The prognosis, according to some authors, is generally favorable; but it seems to me that this opinion should be qualified by the circumstances surrounding the case—first, by the length of time the disease has already existed; second, the general condition of the patient as to hygienic surroundings; third, the condition of the cornea at the time of commencing treatment; fourth, the presence or absence of ulceration of the cornea, and its extent, and the peculiarities of constitution as to a tendency to scrofula, etc., by inheritance. If the cornea present a hazy, milky appearanee, and there be abscess or ulceration, involving to any considerable extent the cornea, I should be very chary in making a favorable prognosis, more especially if the inflammatory action is yet unsubdued, and one, two or more of the conditions just enumerated be present to any considerable extent, which exerts any influence on the probable termination of the disease. If ulceration *does* exist, it cannot be definitely prognosticated that partial staphyloma may not take place as a sequellæ of prolapse of the iris through these ulcerations, or *this* ulceration, if you please. While this may be a result occasionally to be expected, there comes too strabismus, incomplete amaurosis, and central capsular cataract; and surely no gentleman present will attempt to deny that some of these are grave diseases; and they are often just what happens when the disease does not yield to treatment, or when it is neglected.

If the disease yields to treatment, the swelling and tumefaction of the lids gradually diminish, and a somewhat pale, livid and wrinkled skin takes the place of the former swollen and shining red. While the redness and chemosis of the conjunctiva subsides, the enlarged papillæ of the palpebal conjunctivæ are observable for a longer or shorter time; the purulent discharge gradually diminishes, and the disease yields, with a gradual subsidence of all the symptoms, in proportion to the extent of the corneal ulceration, which insures a slow and tedious convalescence. The disease seems so readily recognized, that it is hardly necessary to speak of differential diagnosis, the disease being essentially the same, no matter what the cause.

The treatment varies but little, no matter whether the affection result from a gonorrhœal or leucorrhœal discharge; and authors seem to have fallen into the habit of using nearly the same remedies, though it is safe to predict that within the next decade of years the old routine will be considerably modified. And let me say here, that I deprecate that course of medication which pretends to cure one disease by the establishment of another a dozen times more severe, especially in so delicate an organ as the eye. And though the results of the old treatment have in many instances seemed to be successful, a milder course, I think, would accomplish the same good, with vastly less pain. Soelberg Wells says the first indication is prevention, which is to be accomplished by washing the eyes with warm water, thoroughly and repeatedly, directly after birth. The towels, sponges, lint, etc., should be perfectly clean, and used for no other purpose, upon which suggestion it would be well often to act. Authors seem to differ somewhat as to the precise articles to be selected for the local inflammation. Jones would use nitrate of silver, from six to ten grains to the ounce of water, and strong red oxide of mercury ointment; while Wells takes a very different view of remedies, and treats his cases with a weak solution of sulphate of zinc, one to two grains to the ounce of water. It is needless to say that I am most decidedly favorable to the latter kind of treatment, the ones enumerated taken as examples. Before the application of any remedy calculated to act upon the local disease, the eye should be *thoroughly* cleansed with tepid water, and sometimes a little milk additional; after which, if the discharge is thin and more or less watery, three or four applications daily of chloride or sulphate of zinc, one to three or four grains to the ounce of glycerin, applied either with a little syringe, or what is perhaps better still, a camel's hair pencil, while the lids at their edges are smeared over with a little simple cerate, sweet oil, or some such substance, to prevent their gluing together, will generally be sufficient. A weak solution of alum is sometimes used for the same purpose, and has been held efficacious. I believe that even in the purulent stage of the discharge, no other remedy than the zinc need be used as an astringent, as its strength, if necessary, may be increased to the desired amount. Before each application, a copious douche with tepid water will be of advantage. When the eyelids and sinuses are swollen and sarcomatous-looking, red and gorged with blood, so that you can see the minute vascular points almost ready to bleed, while the temperature about the eye is elevated, the extraction of a little blood by means of leeches from the temple, or by cupping, (and this should be carefully done, as the systems of children are powerfully affected by the loss of a comparatively small quantity of blood), is demanded. Sometimes, it will be found that the *cold* application is most grateful to the patient, and if it is properly attended to, as Haynes Walton says, so as to prevent undue chilling of the surface, it should be encouraged. Soothing applications seem to me to be eminently proper; and as a local application of this class, the pulverized elm, as a poultice, is generally easily obtained, and withal a good one. Ectropion is a somewhat frequent occurrence during this disease, and should always be reduced by gentle manipulation, if possible, but which cannot sometimes be done, when it will be necessary to scarify the lids slightly, after which they will generally be turned back without much trouble. As to the propriety of constitutional treatment, authors differ—some say yes, others no; yet, the propriety of calomel in minute doses, with quinine, when there is a tendency to corneal ulceration, is recognized by the majority. The nourishment of the little patient should by all means be attended to, and intercurrent affections should receive appropriate treatment.

